# Supplementary material for: The interactome of intact mitochondria by cross-linking mass spectrometry provides evidence for coexisting respiratory supercomplexes
Source: Mol Cell Proteomics. 2017 Dec 8;17(2):216–32. doi: 10.1074/mcp.RA117.000470 (PMC5795388; doi:10.1074/mcp.RA117.000470)
Supplement: Supplemental Data [file supp_RA117.000470_133922_0_supp_23978_fzffwf.zip › spectra_annotation/mito_DR_spectra_annotation/75-1-2-1-14-1.pdf]

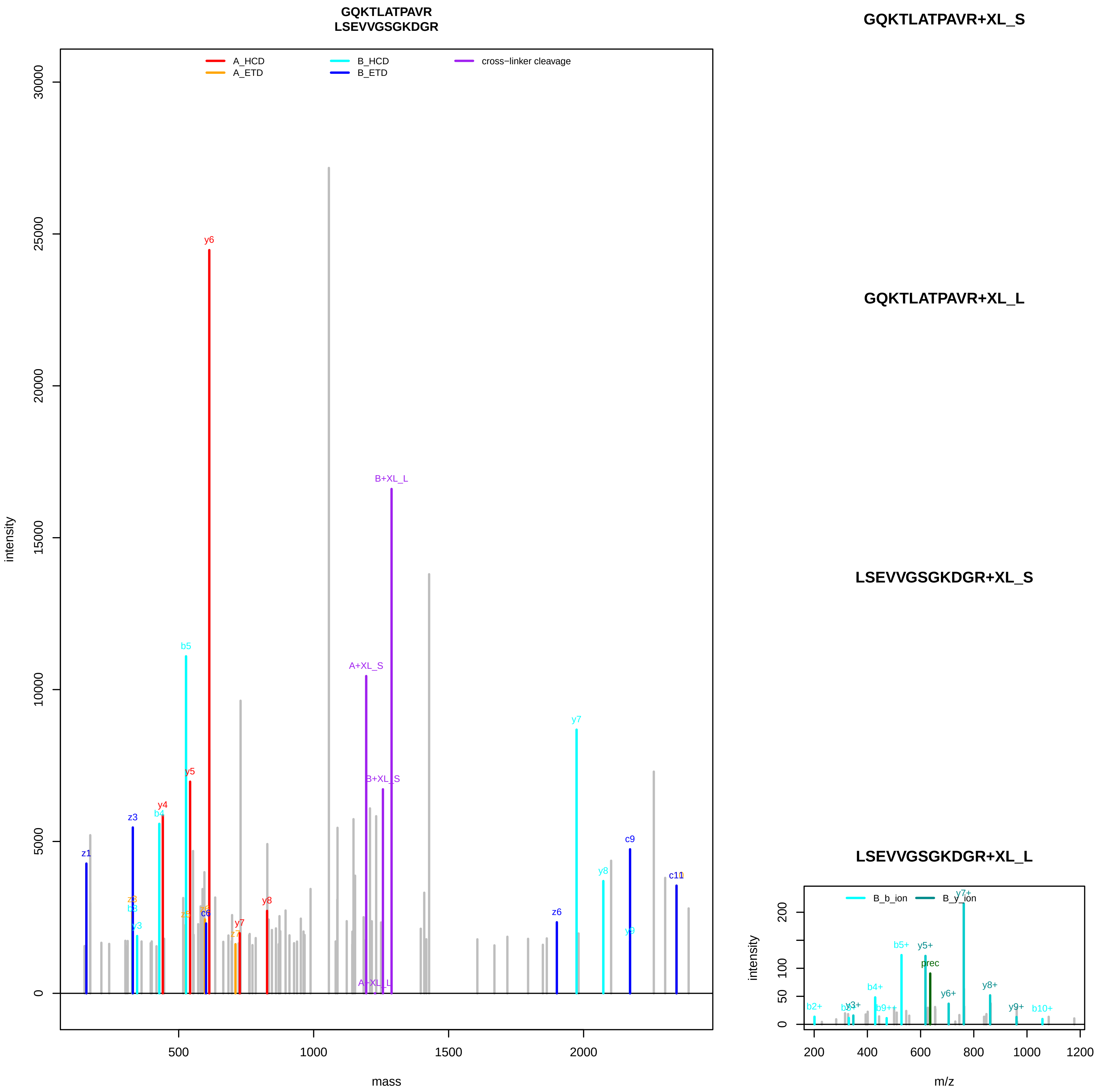

GQKTLATPAVR
LSEVVGSGKDGR
A_HCD
A_ETD
B_HCD
B_ETD
cross−linker cleavage
intensity
mass
y6
B+XL_L
A+XL_S
B+XL_S
A+XL_L
b5
y5
y4
b4
z3
z1
b3
y3
c6
y7
y8
z6
y7
y8
c9
y9
c11
GQKTLATPAVR+XL_S
GQKTLATPAVR+XL_L
LSEVVGSGKDGR+XL_S
LSEVVGSGKDGR+XL_L
B_b_ion
B_y_ion
m/z
b2+
y3+
b4+
b5+
y5+
prec
y6+
y7+
y8+
y9+
b10+
b9++
